# Botulinum toxin dosing in arm muscles: contextual factors

**DOI:** 10.1007/s00702-021-02307-1

**Published:** 2021-01-30

**Authors:** Dirk Dressler, Bruno Kopp, Fereshte Adib Saberi

**Affiliations:** 1 Movement Disorders Section, Department of Neurology, Hannover Medical School, Carl-Neuberg-Str. 1, 30625 Hannover, Germany; 2 Department of Neurology, Hannover Medical School, Hannover, Germany

**Keywords:** Botulinum toxin, Therapy, Dystonia, Spasticity, Writer's cramp, Dosing tables, Contextual factors

## Abstract

Botulinum toxin (BT) has been successfully used for many years to treat various muscle hyperactivity disorders including dystonia and spasticity. Its dosing is guided by dosing tables describing target muscles and dose ranges. To refine the BT dosing, we wanted to analyse how contextual factors may influence the injector's final dosing decision.

In a retrospective review of real-life data of 1170 BT treatments, we studied the influence of various contextual factors on the BT doses in 21 arm muscles of 252 patients receiving BT therapy for different muscle hyperactivity disorders.

We found that BT arm doses are significantly higher in treatment of spasticity than in treatment of dystonia. We also found that spontaneous arm dystonia requires higher BT doses in a proximal application pattern, whereas task specific writer's cramp requires considerably reduced BT doses with a distal application pattern. Injections of non-arm muscles influence the BT dosing in arm muscles only marginally.

Our study demonstrates that BT dosing does not only depend on the particularities of the individual target muscle injected, such as its volume and its static or phasic function. BT dosing and its application pattern rather depend on additional contextual factors such as the aetiology and pathophysiology of the muscle hyperactivity treated. These contextual factors need to be included in dosing tables and may improve the outcome of BT therapy.

## Introduction

Botulinum toxin (BT) has been successfully used for many years to treat various muscle hyperactivity disorders including dystonia and spasticity. As BT acts locally it has to be injected in appropriate doses into the relevant muscles. This dosing is guided by dosing tables so far only describing dose ranges irrespective of specifying contextual factors. To refine BT dosing we wanted to analyse how contextual factors including the aetiology and pathophysiology of the treated condition and additional BT application elsewhere in the body may influence the injector's final dosing decision.

## Methods

### Design

The study is a non-interventional retrospective review of treatment data of patients receiving routine BT therapy of arm muscles. Contextual factors potentially influencing BT arm muscle dosing included (1) the muscle hyperactivity's aetiology (dystonia vs spasticity), (2) its pathophysiology (arm dystonia vs writer's cramp) and (3) additional BT application (BT application for isolated arm spasticity vs BT application for hemispasticity vs BT application for tetraspasticity).

### Data base

Treatment data originated from the computerised BT therapy data base of the Movement Disorders Section, Department of Neurology, Hannover Medical School, Hannover, Germany. This institution was founded 12 years ago by one of the authors (DD) and is specialised in BT therapy. Currently, its annual BT usage is in excess of 20,000 100MU vial equivalents of onabotulinumtoxinA (ONA, Botox®, Allergan, Dublin, Ireland) and incobotulinumtoxinA (INCO, Xeomin®, Merz Pharmaceuticals, Frankfurt/M, Germany). Data used for this study are real-life data routinely collected at this institution. For each indication, treatment data were consecutively collected until pre-set numbers were reached.

### Treatment algorithms applied

BT therapy is based on the algorithms developed during the last 33 years by one of the authors (DD) and his team. This reference centre is able to perform BT therapy with a minimum of economic and legal restrictions thus being able to exploit the full benefit of this therapy. For all patients treated BT therapy is free of costs. Regulatory recommendations on target muscle selection and dosing, total doses, inter-injection intervals and contraindications are modified wherever necessary. Permission to perform quantitative and qualitative off-label use was applied for and generally granted. Total doses of up to INCO 1500MU and INCO inter-injection intervals down to 6 weeks may be applied wherever necessary.

### BT therapy

The standard reconstitution for ONA 100MU vials and INCO 100MU vials uses 2.5 ml 0.9% NaCl/H_2_O. The standard volume per injection site is 0.5 ml (20MU); hence the number of injection sites is mainly determined by the BT doses applied to each target muscle.

### Patients

The study was performed on 80 patients with unilateral arm spasticity, 83 patients with hemispasticity, 29 patients with tetraspasticity, 50 patients with writer's cramp and 10 patients with arm dystonia. Arm dystonia described patients with spontaneous, non-action induced or non-task specific arm dystonia. Writer's cramp describes patients with task specific arm dystonia triggered by writing.

### Statistics

The significance level was set to *α* = 0.05. As 23/29 patients with tetraspasticity showed bilateral arm involvement, the statistical analysis was based on the arm on the more severely affected and, thus, higher dosed side. ONA and INCO doses were converted into equivalent mouse units (MU-E) based on a conversion ratio of 1:1 (Dressler 2009, 2010; Dressler et al. 2012, 2014b, 2018).

## Results

### Patients

Altogether 1170 BT arm muscle treatments in 21 arm muscles of 252 patients were studied. As shown in Table 1, patients with spasticity (arm spasticity, hemispasticity and tetraspasticity) show a male preponderance (*χ*^*2*^ = 10.08, *p* = 0.001), while patients with dystonia (writer's cramp and arm dystonia) do not show a preponderance (*χ*^*2*^ = 0.276, *p* = 0.606). There was no difference detectable in patient age with spasticity and dystonia (*t* = 1.024, *p* = 0.307).

**Table 1:** Patient demographics

Indication	Patient numbers [*n*]	Patient age (M ± SD) [years]	Patient sex [%]	
			Male	Female
Arm spasticity	80	59.1 ± 14.5	65	35
Hemispasticity	83	58.4 ± 14.7	59	41
Tetraspasticity	29	43.8 ± 16.7	59	41
Writer's cramp	50	57.4 ± 13.5	48	52
Arm dystonia	10	37.2 ± 19.7	40	60

*M ± SD* mean ± standard deviation

### Arm muscle dosing under the contextual factor 'additional BT application'

Table 2 shows BT therapy of 1002 arm muscle treatments in 18 different arm muscles of 192 patients when isolated arm spasticity was treated and when hemispasticity and tetraspasticity were treated. The BT arm dose per patient was 388.3 ± 67.8MU-E in isolated arm spasticity, 322.2 ± 141.9MU-E in hemispasticity and 310.7 ± 161.6MU-E in tetraspasticity. Therefore, it was lower when isolated arm spasticity rather than hemispasticity and tetraspasticity were treated, whereas there was no difference when hemispasticity rather than tetraspasticity was treated (overall group difference, *F* (*df* = 2, 189) = 4.648, *p* = 0.011; post hoc tests: arm spasticity vs hemispasticity, *t* = 2.709, *p*_*Holm*_ = 0.022; arm spasticity vs tetraspasticity, *t* = 2.298, *p*_*Holm*_ = 0.045; hemispasticity vs tetraspasticity, *t* = 0.342, *p*_*Holm*_ = 0.733). The number of target muscles treated per patient was 6.5 ± 2.7 in isolated arm spasticity, 5.3 ± 2.4 in hemispasticity and 5.0 ± 2.0 in tetraspasticity. Therefore, it was also smaller when isolated arm spasticity rather than hemispasticity and tetraspasticity were treated, whereas there was no difference when hemispasticity rather than tetraspasticity was treated (overall group difference, *F (df* = 2, 189) = 6.252, *p* = 0.002; post hoc tests: arm spasticity vs hemispasticity, *t* = 3.137, *p* = 0.006; arm spasticity vs tetraspasticity, *t* = 2.673, *p*_*Holm*_ = 0.016; hemispasticity vs tetraspasticity, *t* = 0.407, *p*_*Holm*_ = 0.684).

**Table 2:** Botulinum toxin therapy of arm muscles under the contextual factor ‘additional botulinum toxin therapy'

Indication	BTT for AS	BTT for HS	BTT for TS	Significance	BTT for ALLS
Number of patients treated [*n*]	80	83	29	n/a	192
Number of arm muscles treated [*n*]	518	436	146	n/a	1002
BT arm dose per patient (M ± SD) [MU-E]	388.3 ± 67.8	322.2 ± 141.9	310.7 ± 161.6	AS vs HS: * AS vs TS: * HS vs TS: ns	378.3 ± 186.3
Number of arm muscles treated per patient (M ± SD) [*n*]	6.5 ± 2.7	5.3 ± 2.4	5.0 ± 2.0	AS vs HS: * AS vs TS: * HS vs TS: ns	6.3 ± 3.0

*n/a* not applicable, *ns* not significant, M ± SD mean ± standard deviation, BT botulinum toxin, BTT botulinum toxin therapy, MU-E equivalent mouse unit, *AS* arm spasticity, *HS* hemispasticity, *TS* tetraspasticity, *ALLS* all spasticity

*Significant

### Arm muscle dosing under the contextual factor 'aetiology'

Table 3 shows the BT therapy of 1002 BT arm muscle treatments in 18 different arm muscles of 192 patients with spasticity (arm spasticity, hemispasticity, tetraspasticity), of 128 BT arm muscle treatments in 12 different arm muscles of 50 patients with writer's cramp and of 40 BT arm muscle treatments in 8 different arm muscles of 10 patients with arm dystonia. The BT arm dose per patient in writer's cramp was 70.3 ± 55.3MU-E, in arm dystonia 196.0 ± 150.8MU-E and in spasticity 378.3 ± 186.3MU-E. With this, the BT arm dose per patient in writer's cramp was 19% of the BT arm dose per patient in spasticity and 36% of the one in arm dystonia. Group differences of BT arm dose per patient reached statistical significance (overall group difference, *F* (*df* = 2, 249) = 75.83, *p* < 0.001; post hoc tests: spasticity vs dystonia, *t* = 3.254, *p*_*Holm*_ = 0.003; spasticity vs writer's cramp, *t* = 12.145, *p*_*Holm*_ < 0.001; dystonia vs writer's cramp, *t* = 2.52, *p*_*Holm*_ = 0.012).

**Table 3:** Botulinum toxin therapy of arm muscles under the contextual factor 'aetiology'

Indication	All Spasticity	Writer's cramp	Arm dystonia	Significance
Number of patients treated [*n*]	192	50	10	n/a
Number of arm muscles treated [*n*]	1002	128	40	n/a
BT arm dose per patient (M ± SD) [MU-E]	378.3 ± 186.3	70.3 ± 55.3	196.0 ± 150.8	ALLS vs WC: * ALLS vs AD: * WC vs AD: *
Number of arm muscles treated per patient (M ± SD) [*n*]	6.3 ± 3.0	2.5 ± 1.5	4.4 ± 2.2	ALLS vs WC: * ALLS vs AD: ns WC vs AD: *

*n/a* not applicable, *ns* not significant, *M ± SD* mean ± standard deviation, *BT* botulinum toxin, *MU-E* equivalent mouse unit, *ALLS* all spasticity, *WC* writer's cramp, *AD* arm dystonia

*Significant

The number of arm target muscles per patient was 2.5 ± 1.5 in writer's cramp, 4.4 ± 2.2 in arm dystonia and 5.7 ± 2.6 in spasticity. Group differences of BT number of target muscles per patient reached statistical significance (overall group difference, *F* (*df* = 2, 249) = 36.35, *p* < 0.001; post hoc tests: spasticity vs dystonia, *t* = 1.73, *p*_*Holm*_ = 0.085; spasticity vs writer's cramp, *t* = 8.481, *p*_*Holm*_ < 0.001; dystonia vs writer's cramp, *t* = 2.267, *p*_*Holm*_ = 0.048).

### Arm muscle dosing under the contextual factor 'pathophysiology'

**Fig. 1:**
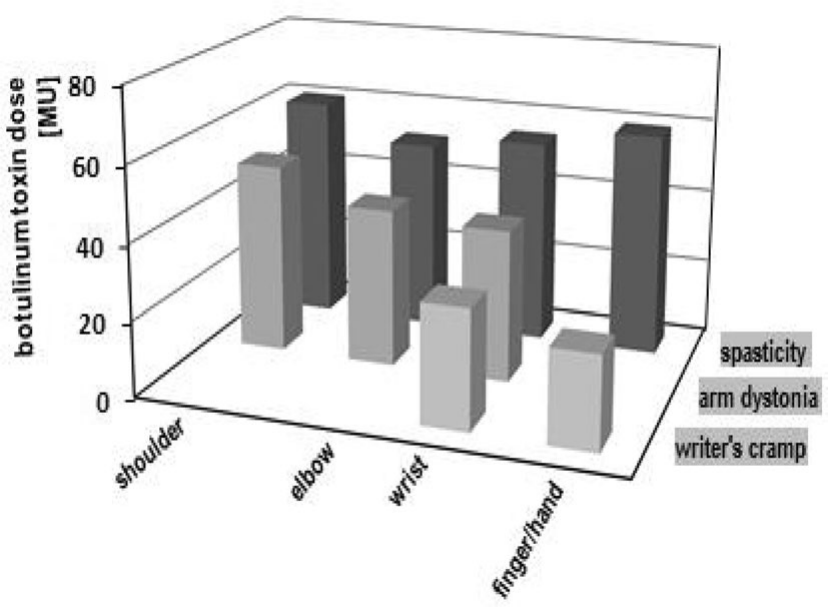
Distribution pattern of botulinum toxin doses in patients with spasticity, arm dystonia and writer's cramps

Table 4 and Fig. 1 show the distribution patterns of BT arm doses in patients with spasticity, arm dystonia and writer's cramps. In the shoulder muscles (M. pectoralis, M. teres major, M. latissimus dorsi, M. deltoideus) BT doses were 60.3 ± 2.1MU-E in spasticity, 50.5 ± 11.9MU-E in arm dystonia and 62.5 ± 53.0MU-E in writer's cramp. The M. deltoideus was occasionally used in writer's cramp to reduce shoulder abduction. In the elbow muscles (M. biceps brachii, M. brachialis, M. brachioradialis and M. triceps brachii) BT doses were 51.5 ± 8.2MU-E in spasticity, 41.9 ± 8.7MU-E in arm dystonia and 30MU-E in writer's cramp. The elbow muscles were very rarely used in the treatment of writer's cramps. In the wrist muscles (M. flexor carpi ulnaris, M. flexor carpi radialis, M. extensor carpi ulnaris and M. extensor carpi radialis) the BT doses were 54.5 ± 5.4MU-E in spasticity, 40.0 ± 0MU-E in arm dystonia and 31.2 ± 12.4MU-E in writer's cramp. The wrist muscles were only rarely used in the treatment of arm dystonia. In forearm muscles and hand muscles (M. pronator teres, M. flexor digitorum superficialis, M. flexor digitorum profundus, M. extensor digirtorum,. M, flexor pollicis brevis, Mm. lumbricales, intrinsic thumb muscles, M. extensor indicis, M. flexor indicis and M. extensor pollicis) the BT dose was 59.4 ± 20.1MU-E in spasticity and 24.4 ± 12.6MU-E in writer's cramp. None of these muscles were targeted in arm dystonia.

**Table 4:** Botulinum toxin therapy of arm muscles under the contextual factor ‘pathophysiology'

Muscle groups	Spasticity *n* = 194	Arm dystonia *n* = 40	Writer's cramp *n* = 128
Shoulder muscles (M ± SD) [MU-E]	*n* = 194 60.3 ± 2.1	*n* = 18 50.5 ± 11.9	*n* = 2 62.5 ± 53.0
Elbow muscles (M ± SD) [MU-E]	*n* = 261 51.1 ± 8.2	*n* = 13 41.9 ± 8.7	*n* = 1 30.0 ± 0
Wrist muscles (M ± SD) [MU-E]	*n* = 160 54.5 ± 5.4	*n* = 9 40 ± 0	*n* = 59 31.2 ± 12.4
Forearm/hand muscles (M ± SD) [MU-E]	*n* = 387 59.4 ± 20.1		*n* = 66 24.4 ± 12.6

*M ± SD* mean ± standard deviation, *N* number, *MU-E* equivalent mouse units

## Discussion

Dosing tables for BT therapy are describing BT doses for the target muscles treated. In their most basic form they give a typical dose, in more elaborate forms they are giving dose ranges. The most recent dosing table is—for the first time—based on an statistical analysis of real-life treatment data (Dressler 2018). It shows typical doses (mean doses), dose variabilities (standard deviations) and dosing limits (minimum, maximum). Also for the first time, it distinguishes target muscle doses according to specific aetiologies of the muscle hyperactivity treated, i.e. spasticity and dystonia. Still, there is a gap to find the final BT dose applied in individual patients. We wanted to fill this gap by studying additional contextual factors influencing the final dosing decision. For this we focussed on BT therapy of arm muscles and studied the dosing effects of various contextual factors.

### Contextual factor 'additional BT therapy'

Although arm muscle doses and number of arm target muscles are reduced when more wide-spread spasticity is treated, this effect is mild and partially non-significant. This may reflect recently introduced changes in treatment algorithms allowing BT high dose therapies in total doses of up to 1250MU of incobotulinumtoxinA (Dressler et al. 2014a; Wissel et al. 2017) thus lifting restrictions of BT total doses hitherto applied.

### Contextual factor 'aetiology'

We found that BT arm doses are significantly higher in treatment of spasticity than in treatment of dystonia. Especially treatment of writer's cramp requires considerably reduced BT dosing. Similar effects are seen in the number of target muscles treated. Reasons for that may include the following: Spasticity includes by definition (Dressler et al. 2017) variable degrees of paresis. When this paresis is substantial and spasticity is strong, robust BT doses may be given without concerns of additional paretic side effects. In contrary, dystonia does not include paresis and concerns about paretic side effects often arise, especially in writer's cramp.

### Contextual factor 'pathophysiology'

We found that spontaneous dystonia ('arm dystonia') and task specific dystonia ('writer's cramp') require a much different BT therapy. Whereas BT therapy of arm dystonia requires a BT distribution patterns with a strong proximal preponderance, the BT distribution pattern for writer's cramp has a strong distal preponderance. This may reflect the typical temporal and geographic expansion of arm dystonia often developing from cervical dystonia. Within this special distribution pattern writer's cramp requires considerably reduced BT doses. This reflects the narrow therapeutic window of distal arm muscles (especially when they are extensors) (Dressler 2000) and the extraordinary complex functionality of finger muscles.

Our study demonstrates that BT dosing does not only depend on the particularities of the individual target muscle injected, such as its volume and its static or phasic function.

BT dosing and its application pattern rather depend on additional contextual factors such as the aetiology and pathophysiology of the muscle hyperactivity treated. BT dosing and its application pattern rather depends on additional contextual factors such as the aetiology and pathophysiology of the muscle hyperactivity treated. These contextual factors need to be included in dosing tables and may improve the outcome of BT therapy. Additional studies into other contextual factors and into other body regions may further refine BT therapy.
